# Bidirectional ventricular tachycardia caused by occlusion myocardial infarction—a case report

**DOI:** 10.1093/ehjcr/ytag055

**Published:** 2026-01-27

**Authors:** Magnus Nossen, Mathis Korseberg Stokke

**Affiliations:** Institute for Experimental Medical Research, Oslo University Hospital, Ullevål Kirkeveien 166, Oslo 0450, Norway; Institute for Experimental Medical Research, Oslo University Hospital, Ullevål Kirkeveien 166, Oslo 0450, Norway; Arrhythmia Unit, Oslo University Hospital, Rikshospitalet, Sognsvannsveien 20, Oslo 0372, Norway

**Keywords:** Bidirectional ventricular tachycardia, Sgarbossa criteria, Occlusion myocardial infarction, Acute coronary syndrome, Arrhythmia, Case report

## Abstract

**Background:**

Bidirectional ventricular tachycardia (BdVT) is an uncommon form of ventricular arrhythmia typically associated with digoxin toxicity or catecholaminergic polymorphic ventricular tachycardia. BdVT in the setting of acute myocardial ischaemia is limited to case reports. We present an example of BdVT caused by occlusion myocardial infarction with original and modified Sgarbossa criteria being positive.

**Case summary:**

An elderly male with known coronary artery disease presented with chest pain. The initial ECG demonstrated a regular wide QRS complex tachycardia with alternating frontal plane axis, consistent with BdVT. No apparent cause of the arrhythmia was identified from the patient's medication history or family history. Detailed ECG analysis revealed that during tachycardia, the QRS complexes exhibited excessively discordant ST segment elevation and depression. ST segment concordance was observed in natively conducted beats of left bundle branch block morphology following termination of the arrhythmia. These findings raised suspicion of occlusion myocardial infarction as the direct cause of BdVT. This was subsequently confirmed by coronary angiography.

**Conclusion:**

BdVT is a distinct form of ventricular tachycardia, characterized by beat-to-beat alternation in QRS axis and morphology. BdVT in the context of acute ischaemia is rare and limited to case reports. Although this clinical presentation is exceptionally rare, in patients with BdVT and symptoms suggestive of acute coronary syndrome, active myocardial ischaemia should be considered as a potential underlying cause. This case highlights the utility of both the original and modified Sgarbossa criteria in identifying acute ischaemia in the setting of a wide complex rhythm.

Learning points

- Bidirectional VT may rarely be caused by acute coronary occlusion. This arrhythmia is difficult to treat and identification and correction of the underlying cause should be top priority.
- Positive original and/or modified Sgarbossa criteria should alert the clinician to the possibility of underlying acute coronary occlusion in wide QRS complexes.

## Introduction

Bidirectional ventricular tachycardia (BdVT) is a distinct form of ventricular arrhythmia characterized by beat-to-beat alternation of QRS axis and morphology. This arrhythmia was initially described in patients with digoxin toxicity. Most reported cases of BdVT occur either as a complication of digoxin therapy or in association with inherited arrhythmia syndromes such as catecholaminergic polymorphic ventricular tachycardia.^1^ Other causes of BdVT include myocarditis,^2^ aconite poisoning,^3^ and hypokalaemic periodic paralysis-induced hypokalaemia.^4^ Its occurrence during acute myocardial infarction is exceptionally rare, with three previously described cases in the litterature.^5–7^ We describe a case of BdVT caused by occlusion myocardial infarction.

## Summary figure

**Figure:**
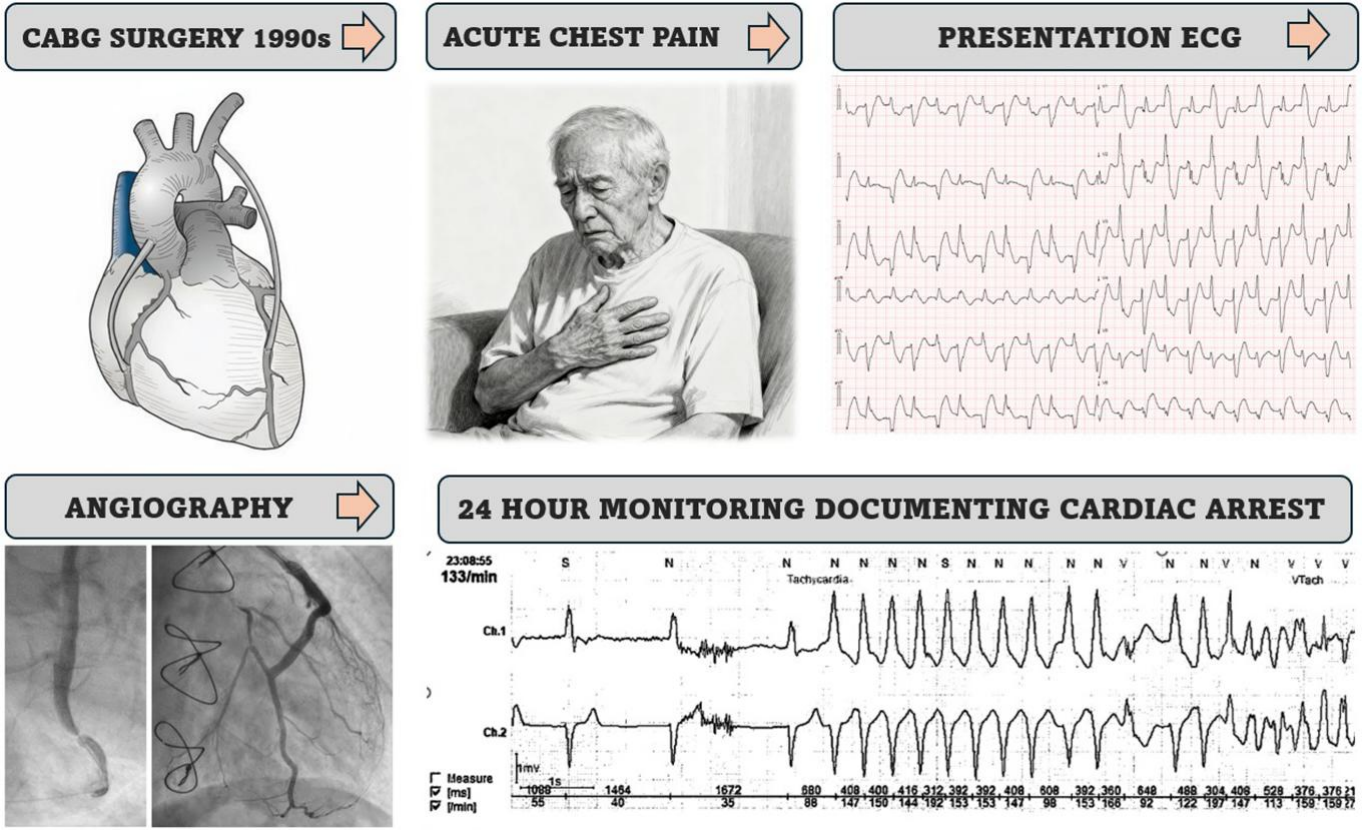
Summary figure depicting timeline and clinical course. The illustration depicting the elderly patient is generated with the use of artificial intelligence.

## Case presentation

An octogenarian male with a history of coronary artery bypass grafting (CABG) surgery in the 1990s, chronic atrial fibrillation, and left bundle branch block (LBBB), contacted emergency medical services (EMS) after experiencing sudden onset chest pain. When EMS arrived, the patient was alert. Heart rate was 125 beats per minute. The blood pressure was 145/85 mmHg, oxygen saturation 95%, and respiratory rate 18 per minute. Lung auscultation revealed fine rales bilaterally. Due to clinical suspicion of acute coronary syndrome, 300 mg acetylsalicylic PO and 2.5 mg morphine IV were administered by the paramedics.

A prehospital ECG recorded during chest pain showed a regular wide complex tachycardia with beat-to-beat alternating frontal plane axis. This arrhythmia persisted for 10 min during transport, then spontaneously terminated.

**Figure 1:**
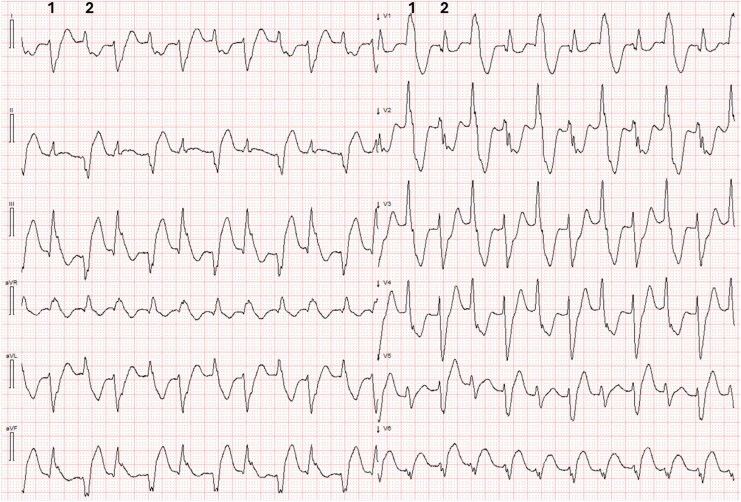
ECG recorded by EMS. Standard lead lay out. Paper speed 25 mm/s. Limb and precordial leads are recorded simultaneously. Numbers 1 and 2 indicate two distinct QRS morphologies.

The ECG in *Figure 1* demonstrates a regular wide-complex tachycardia at 125 bpm. All QRS complexes display a right bundle branch block (RBBB)-like morphology in V1. In the frontal plane, QRS axis alternates with each beat, resulting in two distinct QRS morphologies. The first morphology (1) resembles RBBB with concomitant left posterior fascicular block, while the second morphology (2) resembles RBBB accompanied by a left anterior fascicular block. Both patterns differ from the patient's baseline left bundle branch block.

**Figure 2:**
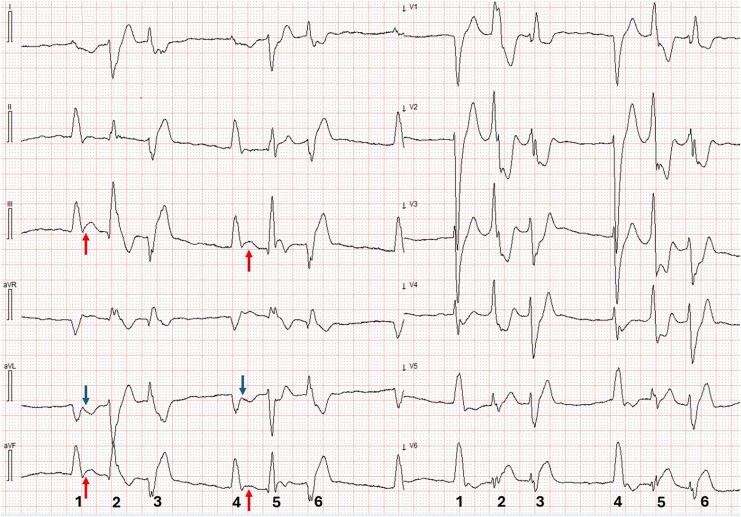
This ECG shows atrial fibrillation with intermittent AV conduction and LBBB (beats 1 and 4). Premature ventricular complexes (PVCs) in the form of ventricular couplets are observed (beats 2–3 and 5–6). QRS axis and morphology of the PVCs are identical to those seen during BdVT. In addition to frequent PVCs, this ECG shows clear signs of ongoing transmural ischaemia. Red arrows point to concordant ST elevation and blue arrows mark concordant ST depression in natively conducted beats of LBBB morphology.

Following arrhythmia termination, another ECG shown in *Figure 2* was recorded en route to the emergency department (ED).

Wide QRS complexes should be followed by ST segment and T wave discordance, i.e. the ST segment and T waves should be of opposite polarity to the preceeding QRS complex. This discordance is expected for wide QRS complexes. In *Figure 2* the LBBB-conducted complexes (beats 1 and 4) have concordant ST segment elevation (leads III and aVF, red arrows) and concordant ST depression (lead aVL, blue arrows). Concordant ST segments satisfy the original Sgarbossa criteria for acute myocardial infarction in LBBB.^8^

According to the modified Sgarbossa criteria, excessively discordant ST elevation or depression (defined as an ST segment elevation or depression that exceeds 25% of the amplitude of the preceding S or R wave) is both specific and sensitive for acute MI in the setting of LBBB and paced rhythm.^9^ The criteria are likely applicable to other wide QRS complexes although not formally validated.

**Figure 3:**
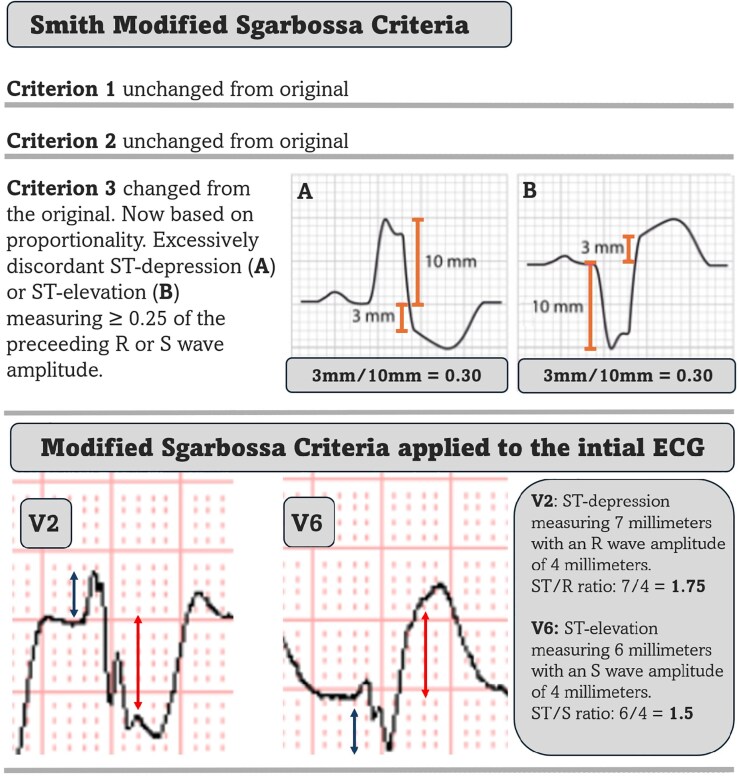
Top showing Smith modified Sgarbossa criteria. Bottom showing lead V2 and V6 from the initial ECG. Blue arrows mark R and S wave amplitude. Red arrows mark ST-segment deviation. There is excessively discordant ST depression in lead V2 and excessively discordant ST elevation in lead V6.

QRS complexes from lead V2 and V6 from the BdVT are displayed at the bottom of *Figure 3*. Careful examination reveals markedly elevated ST/R and ST/S ratios in V2 and V6 (1.5 and 1.75, respectively) which strongly support coronary occlusion as the underlying mechanism of the ventricular arrhythmia.

On arrival at the local ED, the patient’s symptoms had improved. A repeat ECG (not shown) demonstrated atrial fibrillation with LBBB and short runs of polymorphic ventricular tachycardia. The natively conducted LBBB complexes no longer displayed concordant ST elevation, suggesting partial reperfusion. Polymorphic VT in acute coronary syndrome remains an ominous finding with high risk of cardiac arrest. High-sensitivity troponin I was elevated at 237 ng/L (reference <34 ng/L), and the patient was transferred to a PCI-capable centre, where he arrived pain-free.

**Figure 4:**
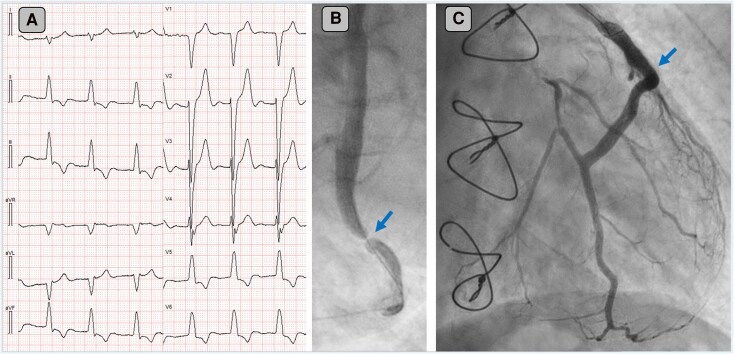
*A*) Repeat ECG before coronary angiography now showing T wave inversions as a sign of reperfusion. Concordant ST segments are no longer present. *B*) Blue arrow indicate subtotal stenosis of venous graft to a large second marginal branch of the LCx. *C*) blue arrow pointing to stented area after PCI.

On arrival at the PCI center, the ECG in *Figure 4A* was recorded. The ECG shows atrial fibrillation with LBBB and signs of reperfusion, evidenced by T wave inversions in the inferolateral leads. Coronary angiography revealed complete native triple-vessel occlusion, a finding not uncommon many years after CABG surgery. The arterial graft to the LAD and the venous grafts to the RCA and a large first diagonal branch were patent. The culprit lesion indicated by the blue arrow in *Figure 4B* was a subtotal (99%) occlusion with TIMI 2 flow in the venous graft to the second marginal branch. A drug-eluting Abbott Xience Pro stent (4.0 × 18 mm) was implanted after lesion preparation with cutting and non-compliant balloons, followed by high-pressure post-dilatation with a 4.5 mm non-compliant balloon to 24 bar. Following PCI, TIMI 3 flow was successfully restored as shown in *Figure 4C* with the blue arrow pointing to the culprit lesion after stent placement.

Echocardiography showed a posterior and lateral wall motion abnormality, with a global left ventricular ejection fraction of 39%. The high-sensitivity troponin I peaked at 40 758 ng/L. Frequent PVCs, but no sustained arrhythmias were observed following revascularization. Contemporary heart failure therapy was initiated with a regimen including a RAAS blocker, mineralocorticoid receptor antagonist, SGLT2 inhibitor, and slow-release metoprolol 75 mg twice daily.

Due to frequent PVCs, a 24-h ambulatory Holter monitor was conducted four weeks after the myocardial infarct, revealing 6955 PVCs, 23 triplets, and three episodes of non-sustained ventricular tachycardia, each consisting of five consecutive ventricular beats. The average heart rate was 42 bpm, and the patient reported significant exercise intolerance. Consequently, the beta-blocker dose was reduced to 75 mg daily, with a follow-up Holter monitoring scheduled to reassess ventricular ectopy and heart rate. Following adjustment of the beta-blocker dosage, the patient reported improved well-being and reduced exercise intolerance.

**Figure 5:**
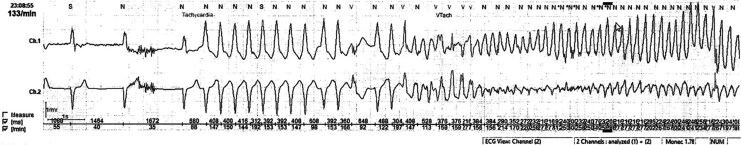
Excerpt from the HOLTER monitoring showing onset of ventricular fibrillation.

A repeat Holter monitor was performed. While wearing the Holter device at home, the patient became unresponsive. *Figure 5* shows an excerpt from the Holter recording with the onset of ventricular arrhythmia causing cardiac arrest being documented. Advanced cardiac life support was commenced and lasted for 45 min, including multiple defibrillation attempts. Despite all efforts, resuscitation was unsuccessful.

## Discussion

BdVT in the context of acute ischaemia is rare and limited to case reports.^5–7^ BdVT secondary to occlusion myocardial infarction has not previously been described. Application of original and modified Sgarbossa criteria for ischaemia diagnosis in a patient with BdVT is not previously referenced in the literature. This case highlights the utility of both the original and modified Sgarbossa criteria in identifying acute ischaemia in the setting of a wide complex rhythm. While occlusion myocardial infarction as a cause of BdVT is exceptionally rare, the presence of BdVT in a patient with symptoms suggestive of acute coronary syndrome should prompt consideration of ongoing myocardial ischaemia as an underlying aetiology.

The arrhythmic mechanism of BdVT is beyond the scope of this case report. However, similar to catecholaminergic polymorphic VT and digoxin toxicity, ischaemia can disrupt calcium handling in cardiomyocytes, leading to delayed afterdepolarizations and the generation of premature ventricular complexes or non-sustained arrhythmias. Sustained BdVT is thought to require a reentrant circuit with two distinct exit sites, producing alternating QRS axis and morphology. Alternatively, the rhythm may arise from triggered activity at two separate myocardial sites without dependence on reentry. This latter explanation aligns with the clinical observation that BdVT is often resistant to electrical cardioversion, emphasizing the importance of identifying and treating the underlying cause.

The arrhythmia seen in our patient was self-limiting. The patient was managed according to current guidelines. Nevertheless, he suffered an out-of-hospital cardiac arrest and could not be resuscitated, which illustrates that ventricular arrhythmia remains unpredictable and cannot be fully prevented, even with optimal evidence-based therapy.

## Lead author biography


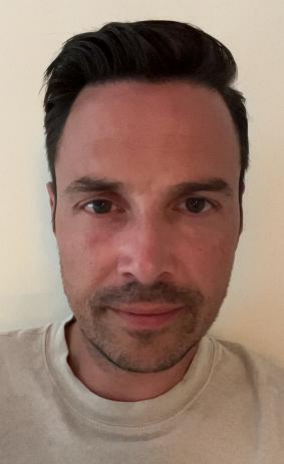


Corresponding author: Magnus Nossen is an MD graduating from the Jagiellonian University Krakow, Poland. Specializing in cardiology and currently a PhD student at the Institute for Experimental Medical Research at University of Oslo and Oslo University Hospital His main areas of interest are the diagnosis of acute coronary syndromes, myocardial ischemia, and the interpretation and management of cardiac arrhythmias

## Data Availability

All relevant information supporting the findings of this case report is included within the article. The underlying data were extracted from the patient’s medical record and are subject to institutional and legal confidentiality regulations; therefore, they are not publicly available and cannot be shared.
